# Optimized sample preparation for two-dimensional gel electrophoresis of soluble proteins from chicken bursa of Fabricius

**DOI:** 10.1186/1477-5956-7-38

**Published:** 2009-10-08

**Authors:** Yongping Wu, Jiyong Zhou, Xin Zhang, Xiaojuan Zheng, Xuetao Jiang, Lixue Shi, Wei Yin, Junhua Wang

**Affiliations:** 1 Key Laboratory of Animal Epidemic Etiology & Immunological Prevention of Ministry of Agriculture, Zhejiang University, Hangzhou 310029, PR China; 2 State Key Laboratory for Diagnosis and Treatment of Infectious Diseases, the First Affiliated Hospital, Zhejiang University, Hangzhou 310003, PR China

## Abstract

**Background:**

Two-dimensional gel electrophoresis (2-DE) is a powerful method to study protein expression and function in living organisms and diseases. This technique, however, has not been applied to avian bursa of Fabricius (BF), a central immune organ. Here, optimized 2-DE sample preparation methodologies were constructed for the chicken BF tissue. Using the optimized protocol, we performed further 2-DE analysis on a soluble protein extract from the BF of chickens infected with virulent avibirnavirus. To demonstrate the quality of the extracted proteins, several differentially expressed protein spots selected were cut from 2-DE gels and identified by matrix-assisted laser desorption ionization time-of-flight mass spectrometry (MALDI-TOF MS).

**Results:**

An extraction buffer containing 7 M urea, 2 M thiourea, 2% (w/v) 3-[(3-cholamidopropyl)-dimethylammonio]-1-propanesulfonate (CHAPS), 50 mM dithiothreitol (DTT), 0.2% Bio-Lyte 3/10, 1 mM phenylmethylsulfonyl fluoride (PMSF), 20 U/ml Deoxyribonuclease I (DNase I), and 0.25 mg/ml Ribonuclease A (RNase A), combined with sonication and vortex, yielded the best 2-DE data. Relative to non-frozen immobilized pH gradient (IPG) strips, frozen IPG strips did not result in significant changes in the 2-DE patterns after isoelectric focusing (IEF). When the optimized protocol was used to analyze the spleen and thymus, as well as avibirnavirus-infected bursa, high quality 2-DE protein expression profiles were obtained. 2-DE maps of BF of chickens infected with virulent avibirnavirus were visibly different and many differentially expressed proteins were found.

**Conclusion:**

These results showed that method C, in concert extraction buffer IV, was the most favorable for preparing samples for IEF and subsequent protein separation and yielded the best quality 2-DE patterns. The optimized protocol is a useful sample preparation method for comparative proteomics analysis of chicken BF tissues.

## Background

Two-dimensional gel electrophoresis (2-DE) is a popular and quite powerful way to separate proteins for proteomics analysis [1]. To date, in the field of avian biology, only a few studies have used proteomics approaches coupling 2-DE and MS to investigate muscle growth and development [2], egg production [3], facial development [4], embryogenesis [5-7], chicken ocular development [8,9] and chicken serum [10]. However, little information about the 2-DE applied to avian bursa of Fabricius (BF), as a central immune organ, is available. BF provides a microenvironment for differentiation and maturation of lymphocytes, particularly B cells [11-15]. The importance of B cells to immunity was first demonstrated using the bursa [16], and the bursa remains an important accessible model for immunity [17] and cancer research [18]. At present, a number of avian diseases, such as the highly pathogenic avian influenza, are a great threat to developing poultry industry and a public health concern. Therefore, it is important to analyze the protein contents of chicken BF that may provide insight into immune regulation. Recently, McCarthy and colleagues used differential detergent fractionation-multidimensional protein identification technology to study the avian BF [19]. Unfortunately, the shortcoming of this method lies in cross-contamination between individual fractions and in the fact that it may be relatively complicated the handle [20]. However sample preparation is critical for detailed visualization of 2-DE profiling and improved insight into biological processes. Tissue contaminants (proteases, lipids, nucleic acids and a broad array of secondary metabolites, etc.) can cause problems such as smearing and horizontal and vertical streaking in 2-DE images [21]. Therefore, maximizing the solubility and recovery of a protein species from a complex mixture is a challenging issue and one that ultimately determines the success of the 2-DE technique. Substantial efforts have been devoted to optimizing the preparation and handling of biological samples in order to enhance the quality of two-dimensional (2-D) gels [22-28], but since the diversity of tissue organization and protein content affect protein solubility, sample preparation must be optimized on a case-by-case basis [29].

Here, the present study was focused to improve the performance and resolution of 2-DE of soluble proteins from chicken BF. We optimized several parameters affecting 2-DE quality, including extraction buffers, lysis conditions, and freezing of immobilized pH gradient (IPG) strips, to obtain consistently well-separated protein profiles. Additionally, we tested the optimized protocol for 2-DE of avibirnavirus-infected chicken BF and the suitability for MS analysis was evaluated. Finally, our protocol was further tested on spleen and thymus tissues from uninfected chickens.

## Results

### Optimization of protein extraction methods

Sample preparation prior to IEF is an important step for separation of proteins from a complex sample in 2-DE. Using chicken BF tissues, which contain many contaminants that strongly interfere with 2-DE, resulting in streaking and smearing, we optimized a protocol for protein extraction and 2-DE. The total amount of extracted BF protein varied, depending on the lysis protocol used. Overall, method A gave 65.8 ± 11.2 mg protein/g tissue, method B, 63.4 ± 14.6 mg/g, and method C, 97.1 ± 6.9 mg/g (means ± SD). Of the three protocols tested, method C resulted in the highest protein yield and the least variation between three replicates.

**Figure 1:**
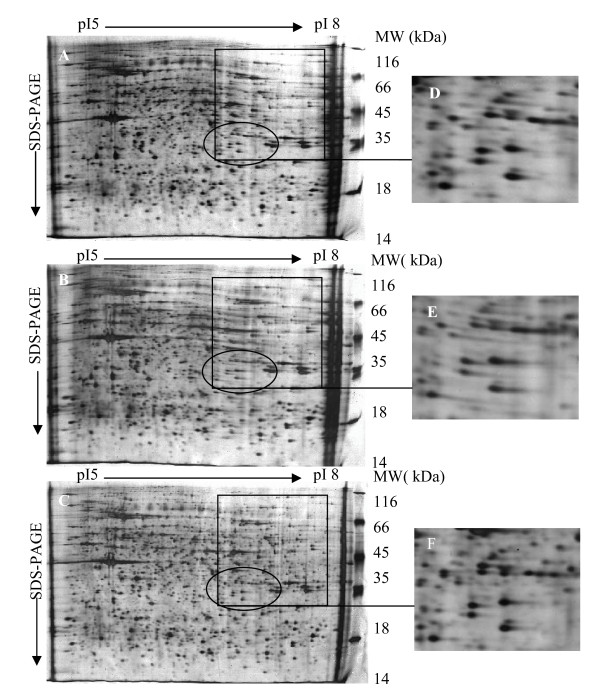
**Two-dimensional gel electrophoresis analysis of soluble bursa of Fabricius proteins extracted by different procedures**. **(A) **sonication extraction. **(B) **vortex extraction **(C) **sonication and vortex extraction. Rectangle areas indicate protein spots that were better resolved by combining sonication and vortex extraction. **(D-F) **illustrates magnifications of the circle in **(A-C)**, respectively.

Three different sample preparation procedures were further compared, and the results are shown in Fig. 1. Three protein migration patterns looked similar with most proteins ranging in size from 14 to 116 kDa. Sample preparation by methods A or B lead to horizontal streaking (rectangles, Fig. 1A and 1B), which indicates protein aggregate formation during the IEF and poor resolubilization and separation in the second gel dimension. This horizontal streaking was not observed in samples prepared with method C (Fig. 1C, rectangles). The method C protocol resulted in a good resolution of stained spots and less horizontal streaking. Also, as shown in Fig. 1D, E, and 1F (magnified circular region of Fig. 1A, B and 1C, respectively), the number and spot resolution of protein spots in the 2-D gel was greater in samples prepared using method C. The total number of protein spots on 2-D gels of method A-, method B-, and method C-treated samples were 1953 ± 125, 1902 ± 89 and 2163 ± 95 (mean ± SD), respectively.

### Evaluation of several extraction buffers

**Figure 2:**
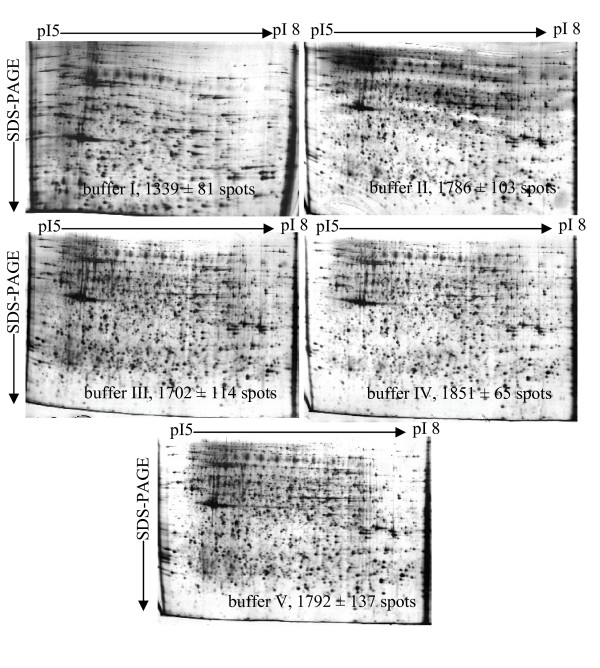
**Optimization of extraction buffers for the solubilization of bursa of Fabricius proteins**. The total number of protein spots detected in gels is buffer I, 1339 ± 81 spots, II, 1786 ± 103 spots, III, 1702 ± 114 spots, IV, 1851 ± 65 spots, and V, 1792 ± 137 spots. The separation of soluble proteins was performed on 24 cm strips over isoelectric point (p*I*) gradient of 5-8. Each gel was loaded with 200 μg of total protein and silver stained.

The goal of tissue homogenization is to solubilize as many proteins as possible to allow for full representation of the proteome. This procedure often requires the use of chaotropic agents, detergents, and reducing reagents. Here, we examined five different extraction buffers for their ability to solubilize proteins during the BF tissue homogenization. As shown in Table 1, buffer I was mainly composed of urea, the zwitterionic detergent 3-[(3-cholamidopropyl)-dimethylammonio]-1-propanesulfonate (CHAPS), carrier ampholytes, and dithiothreitol (DTT). Relative to buffer I, buffer II had additional thiourea, and buffer III contained a lower concentration of CHAPS. The concentration of DTT was lower in buffer IV (50 mM). Buffer V was similar to buffer IV, but with the addition of tris(hydroxymethyl)aminomethane (Tris). As shown in Fig. 2, the number of protein spots detected in gels depended on the extraction buffer: buffer I, 1339 ± 81 spots, II, 1786 ± 103 spots, III, 1702 ± 114 spots, IV, 1851 ± 65 spots, and V, 1792 ± 137 spots (Fig. 2). Buffer I yielded the lowest number of detectable protein spots and visible horizontal streaking, buffer IV yielded the most detectable proteins and slight horizontal streaking. Compared to buffers II and III, buffers IV and V, with less CHAPS and DTT, respectively, had more protein spots in 2-DE, but the 40 mM Tris in buffer V does not significantly improve the quality of 2-D electrophoretograms. Overall, buffer IV performed well as a low cost extraction buffer and was used in further sample preparations.

**Table 1:** Component of five different extraction buffers

**Extraction buffer**	**Components^*a*^**
extraction buffer I	9 M urea, 4% (w/v) CHAPS, 65 mM DTT, 0.2% Bio-Lyte 3/10, 1 mM PMSF, 20 U/ml DNase I, 0.25 mg/ml RNase A

extraction buffer II	7 M urea, 2 M thiourea, 4% (w/v) CHAPS, 65 mM DTT, 0.2% Bio-Lyte 3/10, 1 mM PMSF, 20 U/ml DNase I, 0.25 mg/ml RNase A

extraction buffer III	7 M urea, 2 M thiourea, 2% (w/v) CHAPS, 65 mM DTT, 0.2% Bio-Lyte 3/10, 1 mM PMSF, 20 U/ml DNase I, 0.25 mg/ml RNase A

extraction buffer IV	7 M urea, 2 M thiourea, 2% (w/v) CHAPS, 50 mM DTT, 0.2% Bio-Lyte 3/10, 1 mM PMSF, 20 U/ml DNase I, 0.25 mg/ml RNase A

extraction buffer V	7 M urea, 2 M thiourea, 2% (w/v) CHAPS, 50 mM DTT, 40 mM Tris, 0.2% Bio-Lyte 3/10, 1 mM PMSF, 20 U/ml DNase I, 0.25 mg/ml RNase A

^*a*^CHAPS, 3-[(3-cholamidopropyl)-dimethylammonio]-1-propanesulfonate; DTT, dithiothreitol; Tris, tris(hydroxymethyl)aminomethane; DNase I, Deoxyribonuclease I; RNaseA, Ribonuclease A; PMSF, Phenylmethylsulfonyl fluoride

### Frozen versus non-frozen IPG strips after IEF

**Figure 3:**
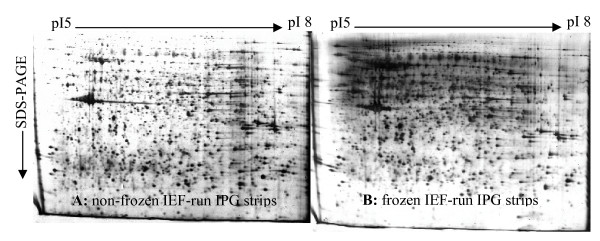
**Soluble bursa of Fabricius proteins, from isoelectric focusing-run immobilized pH gradient strips, directly applied for the second dimension (A) without and (B) with prior freezing at -70°C**. Buffer IV was used to prepare the soluble proteins.

Freezing of IPG gel strips is postulated to increase protein resolubilization and to improve the resolution of 2-DE [29]. In this study, we compared the effect of freezing and non-freezing of IPG strips (three replicate samples for each group) on the second gel dimension. Our results showed that non-frozen and frozen IPG gels resulted in protein expression profiles of very similar quality and with a similar number of spots: 1563 ± 144 for non-frozen IPG strips and 1549 ± 97 for frozen IPG strips. However, strong background was visualized on images of gels from frozen strips (Fig. 3A and 3B). In addition, more horizontal streaking was observed in frozen IPG gels than in gels from non-frozen strips.

### Bursa proteomic analysis of chickens infected with virulent avibirnavirus

**Figure 4:**
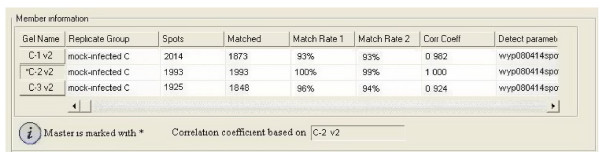
**Statistical match evaluating of three separately-run 2-DE maps**.

**Figure 5:**
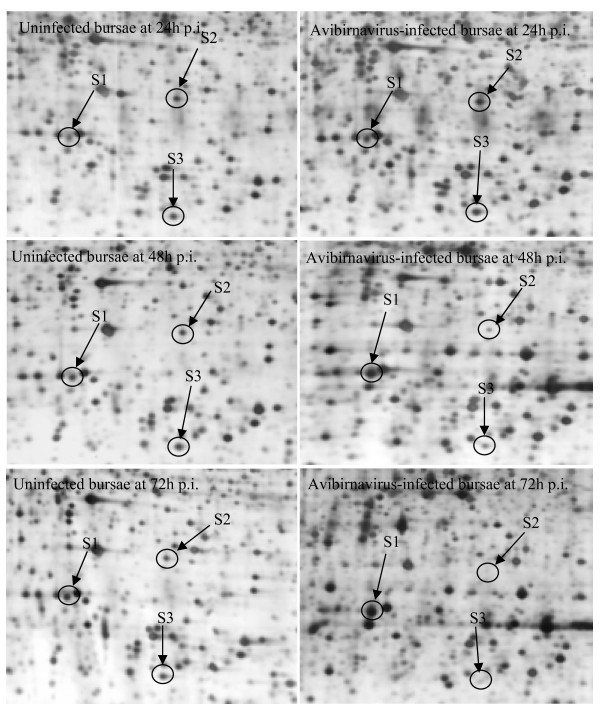
**Two-dimensional gel electrophoresis analysis of chicken bursa infected with virulent avibirnavirus**: comparison of close-ups of two-dimensional patterns of avibirnavirus-infected and uninfected bursae at 24, 48 and 72 h post-inoculation (p.i.).

After optimization of the 2-DE sample preparation methodology, we analyzed BF protein profiles (at various time points) of chickens infected with virulent avibirnavirus and uninfected chickens to find differentially expressed proteins. Reproducibility is important for 2-DE, and matching rate of protein spots is an important parameter for reproducibility of 2-DE. In this study, spots matching (Fig. 4) among the biological triplicate showed well reproducibility using the optimized sample preparation procedure in 2-DE maps of uninfected BF at 24 h post-infection (p.i.). As shown in Fig. 4, the number of protein spots detected in gels is 2014, 1993 and 1925 in triplicate, respectively. The average matching rate is about 96% and correction factor >90%. The results show that the optimized protocol can provide reliable and highly reproducible data. When the gels generated from the avibirnavirus-infected and uninfected bursae were compared, there were many striking differences and three differentially expressed protein spots were selected randomly to be analyzed. Fig. 5 shows that, in the avibirnavirus-infected BF, S1, S2 and S3 protein spots changed remarkably from 24 to 72 h p.i. relative to the control. The data of three protein spots, identified by matrix-assisted laser desorption ionization time-of-flight mass spectrometry (MALDI-TOF MS), was shown in Table 2.

**Table 2:** Putative identification of protein spots in chicken bursa of Fabricius extracted with method C and extraction buffer IV

**Spot No.^*a*^**	**Protein Name**	**Accession** **No.^*b*^**	***M*r (kDa)** **(pred/exp)^*c*^**	**p*I*** **(pred/exp)**	**Matched/** **unmatched^*d*^**	**Protein score/best ion score^*e*^**	**Peptides Identified**
S1	Endoplas-mic reticulum protein ERp29	gi| 67476967	25.36/25.18	7.66/5.34	4/61	132/110	ILEQGEEFAANEVVR

S2	lamin B2	gi| 45384202	67.90/21.79	5.31/7.00	22/77	207/60	IKDLEVLFHR

S3	Proteas-ome 26S subunit, non-ATPase, 14	gi| 74004398	27.13/32.3	6.12/6.65	8/60	96/51	LINANMMVLGHEPR

^*a *^Spot No. is the unique number which refers to the labels in Fig. 5.

^*b*^Accession no. is the MASCOT results of MALDI-TOF/TOF searched from the NCBInr database.

^*c*^pred/exp, predicted/experimental.

^*d*^The number of peaks which match/unmatch to the trypsin peptides.

^*e*^Protein score (based on combined MS and MS/MS spectra) and best ion score (based on MS/MS spectra) were from MALDI-TOF/TOF identification. The proteins had statistically significant protein score of greater than 72 (*p *≤ 0.05) were considered successfully identified.

### Application of the optimized sample preparation protocol to chicken spleen and thymus

**Figure 6:**
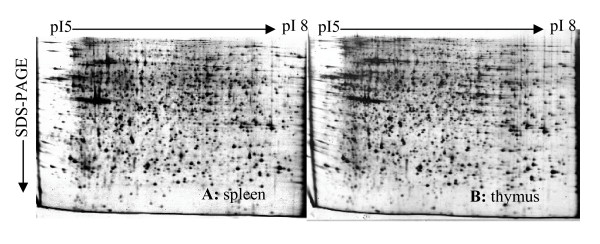
**Application of the optimized protocol for protein extraction from (A) chicken spleen and (B) thymus**. Separation was performed on 24 cm strips over an isoelectric point (p*I*) gradient of 5-8.

The optimized protocol was applied to other immune organs of chicken (spleen and thymus) and the protein yields from the spleen and thymus were 57.1 ± 7.7 and 77.0 ± 5.1 mg protein/g tissue, respectively. The 2-DE protein expression profiles of the spleen and thymus extracts were examined (Fig. 6A and 6B, respectively). Fig. 6 shows that use of this protocol facilitated the extraction of high-quality protein samples suitable of electrophoretic analysis, and the overall quality of the protein profiles was good, with less vertical and horizontal streaking and smearing. The average number of protein spots was 1608 ± 62 for the spleen and 1712 ± 33 for the thymus.

## Discussion

### Optimization of protein extraction methods

We optimized a 2-D gel electrophoresis protocol for chicken BF. We also eliminated the widely-used TCA/acetone precipitation step; though it eliminates instantly proteolytic and other modifying enzymes [30], it can hinder resolubilization of precipitated proteins [31]. We simplified the protein extraction procedure, as fewer steps can minimize protein loss. For our optimization, chicken BF tissues were pulverized into a fine powder under liquid nitrogen to minimize proteolysis and other modes of protein degradation. Next, three different extraction protocols (methods A, B, and C) were tested; protein yields from method C were higher than those from methods A or B. The combination of sonication and vortex in method C seems to increase protein solubility during extraction, which leads to better yields.

2-DE was also used to validate the quality and quantity of the protein extractions. Gels of samples generated by method C (Fig. 1C and 1F) had more well-resolved polypeptide spots and less horizontal streaking than those from methods A or B (Fig. 1A, B, D and 1E). Therefore, method C can better extract BF proteins and is better suited to obtain the quantity and quality of proteins needed for proteome analysis. The possible explanation was that sonication coupling with vortex improved the release and solubility of cellular proteins in method C.

### Evaluation of several extraction buffers

Urea is the standard chaotrope used in 2-DE, typically at concentrations of 5-9.5 M, to disrupt hydrogen bonding and cause protein unfolding and denaturation [32,33]. However, it is not ideal for solubilization of all protein classes, particularly membrane or other hydrophobic proteins [34]. Rabilloud et al [35] introduced the use of the chaotropic reagent thiourea which, in combination with urea, remarkably increased the solvent capacity of the extraction buffer relative to urea alone. The recommended concentration of thiourea is 2 M [35], because a concentration greater than 2 M can inhibit SDS-binding in the second gel dimension [36]. Our study has confirmed that the addition of thiourea raises the solvent capacity of extraction buffer II relative to extraction buffer I (Fig. 2), which contained no thiourea. In the fraction prepared with buffer II, 1786 ± 103 spots were detected, whereas that prepared with buffer I had only 1139 ± 81 spots. Using thiourea in IPG gels, however, can occasionally cause horizontal streaking and/or increases the background of the 2-D gel image when visualized by silver staining, especially in the high molecular weight protein region [29].

The sulfobetaine CHAPS, which aids in protein solubility and minimizes protein aggregation, is commonly used as a detergent for 2-DE [37] in concentrations ranging from 0.5% to 4%. DTT, a reducing reagent, also aids in protein solubility by disrupting intramolecular and intermolecular disulfide bonds [34,37]. We optimized the concentration of CHAPS and DTT in the protein extraction buffer. Higher concentrations of CHAPS and DTT did not improve the 2-DE pattern, and we chose to use 2% CHAPS and 50 mM DTT to help minimize the cost (Fig. 2). Furthermore, although addition of Tris yielded clear 2-DE patterns in the samples prepared with extraction buffer V, it was omitted due to a lack of obvious advantage and increased cost and sample handling. Our final buffer contained protease inhibitors (PMSF) to provide protection from proteolysis and Deoxyribonuclease I (DNase I)/Ribonuclease A (RNase A) to eliminate nucleic acids in our samples.

### Frozen versus non-frozen IPG strips after IEF

We have shown that not only does freezing of IPG gel strips after IEF not enhance protein solubility or improve the resolution of 2-DE, but that images with frozen IPG gel strips had stronger background signals than images with non-frozen strips. This observation is contrary to previously reported results [29]. The stronger background of frozen IPG gel strips may be due to the porous structure of frozen IPG gels, which facilitates the release of thiourea and DTT into the equilibration buffer but may also increase the risk of a high background with silver staining [26,38].

## Conclusion

In summary, lysis method C and subsequent solubilization with extraction buffer IV was the best protocol in our study based on protein extraction efficiency and the good quality of the 2-DE patterns. Correspondingly, when method C and extraction buffer IV were used, good qualities of 2-D gel electrophoresis were also generated in samples from bursa infected with virulent avibirnavirus (Fig. 5), as well as from chicken spleen and thymus tissues (Fig. 6). Moreover, the successful identification of protein spots by MALDI-TOF/TOF MS demonstrated that our optimized extraction protocol was suitable to obtain the quality of proteins required for proteomic studies of chicken BF tissue.

## Methods

### Tissue collection

30-day-old, specific pathogen-free (SPF) Leghorn chickens, purchased from Beijing Merial Vital Laboratory Animal Technology Co. Ltd., were sacrificed by intravenous barbiturates. The BF, spleen and thymus were rapidly excised, rinsed with ice cold PBS (pH 7.2), and immediately frozen in liquid nitrogen.

### Chemicals

All chemicals used were of the highest grade available and were purchased from Bio-RAD (California, USA) and GE-Healthcare (formerly Amersham BioScience, Baie-d'Urfié, QC, Canada). ELGA water (Labwater, Lane End, HP14 3BY, UK) was used throughout.

### Extraction protocols

Triplicate samples of bursa were isolated from nine Leghorn chickens as described above. Frozen BF tissue was ground under liquid nitrogen with a pre-chilled mortar and pestle. According to our previous report [39] and the relevant literatures, combined with IPG strip instruction manual (Bio-Rad, ReadyStrip™ IPG Strip Instruction Manual), to construct an optimal protein extraction protocol of chicken BF tissues, we designated a lysis buffer consisting of 7 M urea, 2 M thiourea, 2% (w/v) CHAPS, 50 mM DTT, 0.2% Bio-Lyte 3/10, 1 mM PMSF, 20 U/ml DNase I, and 0.25 mg/ml RNase A as a sample extraction buffer. One hundred milligrams of ground tissue was directly dissolved in 1.5 ml of extraction buffer and shaken on ice for one hour. Further handling followed one of the following methods:

### Method A

The samples were sonicated for 20 seconds in an ultrasonic bath to improve protein solubility and eliminate contaminants. Sonication consisted of 20 pulses for 1 second each at 200 W. The samples were incubated for 2 seconds on ice at two sonication intervals. The homogenate was then gently shaken on ice for 2 hours and centrifuged at 14, 000 × g at 15°C for 1 hour. These supernatants, labeled as fraction A, were collected and stored as single-use aliquots at -70°C.

### Method B

Instead of sonicating the mixture, it was vortexed vigorously for 2 minutes then cooled for 2 minutes; this sequence was repeated five times and followed by shaking on ice for a further 20-30 minutes. This treatment was repeated four times. The sample was then shaken on ice for 1 hour and clarified by centrifugation at 14,000 × g at 15°C for 1 hour. These supernatants, labeled as fraction B, were stored as single-use aliquots at -70°C.

### Method C

The extract was first sonicated, as in method A, then vortexed as described in method B. Afterwards, the suspension was shaken on ice for 1 hour. Insoluble tissue debris was removed by centrifugation at 14,000 × g at 15°C for 1 hour; the supernatants, labeled as fraction C, were stored as single-use aliquots at -70°C.

### Evaluation of different extraction buffers

Five different extraction buffers (Table 1) were prepared to further test lysis conditions. Their effects were tested after extraction of the BF by method C described above. Briefly, frozen BF tissue was ground under liquid nitrogen with a pre-chilled mortar and pestle. One hundred milligrams of powder were respectively dissolved in 0.75 ml of different extraction buffers, and shaken on ice for 30 minutes. Subsequently the suspension was supplemented the equal volume of the twice concentrated urea solution and shaken on ice for another 30 minutes. Further handling followed the method C.

### Bursa proteomic analysis of chicken infected with virulent avibirnavirus

30-day-old SPF Leghorn chickens were inoculated with virulent avibirnavirus strain NB [40]. At 24, 48, and 72 h p.i., the avibirnavirus-infected and uninfected chicken bursae were harvested for 2-DE analysis, and the soluble proteins were extracted using the final, optimized procedure. All samples were extracted in three independent experiments, after which proteins were separated on 24 cm IPG strips.

### Application of the optimized sample preparation protocol

To determine the optimized sample preparation protocol, chicken spleen and thymus were isolated for 2-DE analysis as described above (three replicate per sample). The soluble proteins were extracted using the final, optimized procedure. All samples were separated on 24 cm IPG strips.

### Protein quantification

Protein concentrations were measured using the Bradford protein assay [41] using bovine serum albumin (BSA) as a standard. The quantification was performed in triplicate sample, and the protein yield was expressed as mean ± standard deviation (SD). After protein quantification, the protein extract was supplemented with a trace of bromophenol blue and was prepared for 2-DE by addition of the appropriate rehydration buffer.

### 2-D gel electrophoresis

Volumes of each fraction containing 200 μg total proteins were loaded for direct comparison of the extraction methods and buffers. The IPG gel strips (pH 5-8 linear gradient, 24 cm, Bio-Rad) were rehydrated overnight in 450 μl rehydration buffer (7 M urea, 2 M thiourea, 2% (w/v) CHAPS, 50 mM DTT, and 0.2% Bio-Lyte 3/10, modified from [39]). Isoelectric focusing (IEF) was carried out using a Protean IEF Cell (Bio-Rad) with a manifold ceramic tray (Bio-Rad) at 20°C for 16 hours at a low voltage (50 V). The rehydrated strips were automatically focused using the following parameters: 500 V, slow, 30 minutes; 1000 V, slow, 30 minutes; 2000 V, linear, 2 hours; 4000 V, linear, 1 hour; 8000 V, rapid, 1 hour; 10,000 V, rapid, 1 hour; 10,000 V, rapid, 90,000 V·H. After IEF, the strips were stored at -70°C overnight. Just prior to second-dimension separation, frozen IPG strips, thawed for 13 minutes at room temperature, and non-frozen IPG strips were incubated for 15 minutes in an equilibration buffer (6 M urea, 20% glycerol, 2% sodium dodecyl sulfate (SDS), 1.5 M Tris, pH 8.8, and 1% (w/v) DTT) and then were incubated for an additional 15 minutes in a modified equilibration buffer, in which DTT was replaced with 2.5% (w/v) iodoacetamide. Two-dimensional electrophoresis was performed on lab-cast 11% SDS-polyacrylamide gel electrophoresis (PAGE) linear gels in a PROTEAN plus Dodeca Cell (Bio-Rad) at 80 V for 45 minutes and then at 200 V until the dye front reached the bottom of the gels.

### Image acquisition and analysis

The 2-D gels were stained by the modified silver staining method compatible with mass spectrometry (MS) [42]. The gels were scanned using the UniscanD3000 scanner (Tsinghua, China), saved as gray-scale TIFF-files, and analyzed using PDQuest 2-D analysis software (Bio-Rad). Image spots were detected using an automatic method and manual corrections. The gel images were normalized according to the total quantity in the analysis set. Relative comparison of intensity abundance between avibirnavirus-infected and uninfected group at three time points was performed using Student's *t *test. Expression intensity ratio value larger than 2.0 were set as a threshold indicating significant changes [39].

### MS analysis and protein identification

Selected protein spots were manually excised from gels and then transferred to V-bottom 96-well plates loaded with 100 μl of 50% ACN/25 mM ammonium bicarbonate solution per well. After being destained for 1 hour, gel plugs were dehydrated with 100 μl of 100% ACN for 20 minutes and then thoroughly dried in a SpeedVac concentrator (Thermo Savant, USA) for 30 minutes. The dried gel particles were rehydrated at 4°C for 45 minutes with 2 μl/well trypsin (Promega, Madison, WI, USA) in 25 mM ammonium bicarbonate, and then incubated at 37°C for 12 hours. After trypsin digestion, the peptide mixtures were extracted with 8 μl extraction solution (50% ACN/0.5% TFA) per well at 37°C for 1 hour. Finally, the extracts were dried under the protection of N2.

The peptides were eluted with 0.8 μl matrix solution (α-cyano-4-hydroxy-cinnamic acid (CHCA, Sigma, St. Louis, MO, USA) in 0.1% TFA, 50% ACN) before spotted on the target plate. Samples were allowed to air-dry and analyzed by 4700 MALDI-TOF/TOF Proteomics Analyzer (Applied Biosystems, Foster City, CA, USA). The UV laser was operated at a 200 Hz repetition rate with wave length of 355 nm. The accelerated voltage was operated at 20 kV. Myoglobin digested by trypsin was used to calibrate the mass instrument with internal calibration mode. All acquired spectra of samples were processed using 4700 Explore™ software (Applied Biosystems) in a default mode. Parent mass peaks with mass range 700-3200 Da and minimum S/N 20 were picked out for tandem TOF/TOF analysis. Combined MS and MS/MS spectra were submitted to MASCOT (V2.1, Matrix Science, London, UK) by GPS Explorer software (V3.6, Applied Biosystems) and searched with the following parameters: NCBInr database, taxonomy of bony vertebrates or viruses, trypsin digest with one missing cleavage, none fixed modifications, MS tolerance of 0.2 Da, MS/MS tolerance of 0.6 Da, and possible oxidation of methionine. Known contaminant ions (keratin) were excluded. MASCOT protein scores (based on combined MS and MS/MS spectra) of greater than 72 were considered statistically significant (*p *≤ 0.05). The individual MS/MS spectrum with statistically significant (confidence interval ≥ 95%) best ion score (based on MS/MS spectra) were accepted. To eliminate the redundancy of proteins that appeared in the database under different names and accession, numbers, the single-protein member belonging to the species *Gallus *or else with the highest protein score (top rank) was singled out from the multi-protein family.

## Abbreviations

2-DE: two-dimensional gel electrophoresis; 2-D: two-dimensional; BF: bursa of Fabricius; IPG: immobilized pH gradient; CHAPS: 3-[(3-cholamidopropyl)-dimethylammonio]-1-propanesulfonate; DTT: dithiothreitol; Tris: tris(hydroxymethyl)aminomethane; DNase I: Deoxyribonuclease I; RNaseA: Ribonuclease A; IEF: isoelectric focusing; SDS: sodium dodecyl sulfate; PAGE: polyacrylamide gel electrophoresis; PMSF: phenylmethylsulfonyl fluoride; pI: isoelectric point; MALDI-TOF MS: matrix-assisted laser desorption ionization time-of-flight mass spectrometry; SPF: specific pathogen free; BSA: bovine serum albumin; p.i.: post infection; MS: mass spectrometry; TCA: trichloroacetic acid.

## Competing interests

The authors declare that they have no competing interests.

## Authors' contributions

YPW designed and executed all experiments, performed the data analysis, constructed the relational database and wrote the manuscript. JYZ was scientific lead and responsible for the experimental design, supervision and writing of the manuscript. XZ and XJZ finished some experiment and provided advice. XTJ and WY conducted sample preparation and analysis. LXS and JHW contributed the sample collection. All authors read and approved the final manuscript.
